# The impact of the novel coronavirus disease (COVID-19) pandemic on drug overdose-related deaths in the United States and Canada: a systematic review of observational studies and analysis of public health surveillance data

**DOI:** 10.1186/s13011-021-00423-5

**Published:** 2021-11-29

**Authors:** Sameer Imtiaz, Frishta Nafeh, Cayley Russell, Farihah Ali, Tara Elton-Marshall, Jürgen Rehm

**Affiliations:** 1 Institute for Mental Health Policy Research, Centre for Addiction and Mental Health, 33 Russell Street, Toronto, Ontario M5S 2S1 Canada; 2 School of Epidemiology and Public Health, Faculty of Medicine, University of Ottawa, 600 Peter Morand Crescent, Ottawa, Ontario K1G 5Z3 Canada; 3 Campbell Family Mental Health Research Institute, Centre for Addiction and Mental Health, 250 College Street, Toronto, Ontario M5T 1R8 Canada; 4 Dalla Lana School of Public Health, University of Toronto, 6th Floor, 155 College Street, Toronto, Ontario M5T 3M7 Canada; 5 Department of Epidemiology and Biostatistics, Schulich School of Medicine and Dentistry, Western University, Kresge Building, London, Ontario N6A 5C1 Canada; 6 Department of Health Sciences, Lakehead University, SN 1006, 955 Oliver Road, Thunder Bay, Ontario P7B 5E1 Canada; 7 Institute of Medical Science, University of Toronto, Room 2374, 1 King’s College Circle, Toronto, Ontario M5S 1A8 Canada; 8 Department of Psychiatry, University of Toronto, 8th Floor, 250 College Street, Toronto, Ontario M5T 1R8 Canada; 9 Institute for Clinical Psychology and Psychotherapy, TU Dresden, Chemnitzer Str. 46, 01187 Dresden, Germany; 10 Department of International Health Projects, Institute for Leadership and Health Management, I.M. Sechenov First Moscow State Medical University, Trubetskaya Str., 8, B. 2, Moscow, Russian Federation 119992; 11 Center for Interdisciplinary Addiction Research (ZIS), Department of Psychiatry and Psychotherapy, University Medical Center Hamburg-Eppendorf (UKE), Martinistraße 52, 20246 Hamburg, Germany

**Keywords:** North America, United States, Canada, SARS-CoV-2, COVID-19, Drug overdose, Death

## Abstract

**Background:**

There are preliminary indications that the trajectory of drug overdose-related deaths in North America has been exacerbated due to the novel coronavirus disease pandemic (COVID-19). As such, the impact of COVID-19 on drug overdose-related deaths was examined through a systematic review of the literature and percentage change analyses of surveillance data.

**Methods:**

Systematic searches in electronic databases were conducted, a topical issue brief and bibliography were reviewed, reference lists of included studies were searched and expert consultations were held to identify studies (Registration # CRD42021230223). Observational studies from the United States and Canada were eligible for inclusion if drug overdose-related deaths were assessed in quantitative or qualitative analyses onwards from at least March 2020. In addition, percentage changes comparing drug overdose-related deaths in the second annual quarter (Q2 2020 [April to June]) with the first annual quarter (Q1 2020 [January to March]) were generated using national and subnational data from public health surveillance systems and reports from jurisdictions in the United States and Canada.

**Results:**

Nine studies were included in the systematic review, eight from the United States and one from Canada. The maximum outcome assessment period in the included studies extended until September 2020. Drug overdose-related deaths after the onset of COVID-19 were higher compared with the months leading up to the pandemic in 2020 and the comparative months in 2019. In additional percentage change analyses, drug overdose-related deaths increased by 2 to 60% in jurisdictions in the United States and by 58% in Canada when comparing Q2 2020 with Q1 2020.

**Conclusions:**

Drug overdose-related deaths increased after the onset of COVID-19. The current situation necessitates a multi-pronged approach, encompassing expanded access to substance use disorder treatment, undisrupted access to harm reduction services, emphasis on risk reduction strategies, provision of a safe drug supply and decriminalization of drug use.

**Supplementary Information:**

The online version contains supplementary material available at 10.1186/s13011-021-00423-5.

## Introduction

The severe acute respiratory syndrome coronavirus (SARS-CoV-2) is a zoonotic coronavirus, the causative agent of the novel coronavirus disease (COVID-19) [1]. Given the severity of the outbreaks across the globe, the World Health Organization declared it a pandemic on March 11, 2020. More than 119 million confirmed cases and 2.6 million deaths have been documented as of March 15, 2021, with about 28% of them occurring within North America [2]. In response to the rising burden of disease, government authorities have implemented public health measures to reduce the contact rate, such as physical distancing restrictions, closures of non-essential services, working from home and limitations on public gatherings and events [3]. Self-isolation after potential exposure or symptom development has been recommended or mandated depending on the local jurisdiction. Despite their utility in reducing the spread of the disease, these measures are associated with substantial social and economic consequences [3, 4], with disproportionate impacts on marginalized populations, including people who use drugs (PWUD).

Prior to the declaration of the pandemic in March 2020, the United States and Canada were in the midst of a drug overdose crisis [5, 6], which has worsened since the onset of COVID-19. “Big events” – major disruptions that create social instability (e.g. natural disasters, financial crises and heroin shortages) – disrupt drug market dynamics, resulting in changes to the availability, accessibility, price and potency of drugs, ultimately leading to the switching of drugs and alteration of consumption habits among PWUD [7]. Indeed, increases in the price and adulteration of drugs and decreases in the availability of drugs have been documented since the onset of COVID-19 [8], with subsequent engagement in riskier behaviors (e.g. sharing paraphernalia) by PWUD [9]. Simultaneous to the changes in drug market dynamics, there have been interruptions in the delivery of harm reduction and addiction treatment services due to COVID-19 [10]. Experiences of detrimental access and disrupted treatment have been documented for a range of services among PWUD, including supervised consumption services, needle exchange programs, mobile outreach programs, withdrawal management services and opioid agonist treatment [10]. Importantly, physical distancing restrictions intended to reduce the transmission of COVID-19 have resulted in drug usage alone by PWUD, limiting the opportunity of others to respond in an event of a drug overdose [9]. Therefore, the risk of drug overdose-related deaths is magnified.

Syndromic surveillance lends support to these findings [11, 12]. Based on the Centers for Disease Control and Prevention’s National Syndromic Surveillance Program in the United States, rates of emergency visits for drug overdoses and opioid overdoses decreased after the initial implementation of the public health measures, but subsequently rebounded starting at the end of March 2020 [11]. Importantly, median emergency visits for drug overdoses and opioid overdoses were higher between March 2020 to October 2020 compared with between March 2019 to October 2019 [11]. A similar pattern is also emerging for drug overdose-related deaths, as two rapid reviews conducted during the early stages of the pandemic suggest increases in drug overdose-related deaths in Canada [13, 14]. However, given the absence of national, real-time mortality statistics, the full extent of the impact of COVID-19 on drug overdose-related deaths remains to be characterized. Reports from multiple jurisdictions can be triangulated to inform interventions and policies in the interim. Accordingly, the present objectives were as follows:

1. Review observational studies that examined the impacts of COVID-19 on drug overdose-related deaths in the United States and Canada.
2. Quantify percentage changes in drug overdose-related deaths before and after the onset of COVID-19 based on public health surveillance systems and reports from the United States and Canada.

## Methods

### Drug overdose-related death case definition

Drug overdose-related deaths hereunder were operationalized as drug poisoning deaths attributable to, but not limited to, opioids, cocaine and amphetamines, which encompassed different types of drugs (pharmaceutical, non-pharmaceutical [i.e. illegal]), places of death (residence, public place, unknown) and manners of death (unintentional, intentional, undetermined), as available.

### Systematic review

The reporting of the systematic review was consistent with the Preferred Reporting Items for Systematic Reviews and Meta-Analyses (PRISMA) Guidelines (Prospero Protocol Registration: CRD42021230223; See reporting checklist in Table S1 in Additional File 1) [15]. Electronic searches were conducted in Medline, Embase and PsycInfo to identify studies published from database inception to December 18, 2020 (see Additional File 1 for the search strategies). In order to be as comprehensive as possible, and to reduce the risk of publication bias, additional sources were drawn upon to identify studies: [1] keyword searches in Google Scholar were conducted [2], an issue brief listing reports of drug overdose-related deaths in the United States was reviewed [16], [3] a bibliography of published studies on addiction-related topics and COVID-19 was assessed [17], [4] reference lists of included studies were examined and [5] expert consultations were held [18]. The titles and abstracts were reviewed in the first round of screening, and the full texts were reviewed in the second round of screening. The selection criteria were as follows:

1. Observational design.
2. Assessments incorporating data onwards from at least March 2020 (considered to be the onset of COVID-19).
3. United States, Canada or Mexico as locations. However, as no studies were found that originated from Mexico, the protocol was modified to include United States and Canada as locations.
4. Drug overdose-related deaths as an outcome.
5. Quantitative or qualitative analyses.

No language restrictions were applied, and unpublished literature was eligible for inclusion. Unpublished literature was included to capture all available evidence, as peer-reviewed manuscripts were expected to be limited in number due the timeliness of the topic. Prior reviews, animal studies, newspaper articles and opinion pieces (i.e. lacking empirical data; e.g. commentaries and research letters) were excluded. Similarly, routine public health surveillance reports were excluded (e.g. [19]), as they tended to include statistics on drug overdose-related deaths rather than quantify the impacts of COVID-19 on drug overdose-related deaths. However, data from them were extracted for the Percentage Change Analyses. Studies that met the selection criteria proceeded to the data extraction, where details on location, time period, population, outcome definition and ascertainment and main findings were noted. Risk of bias assessments were not conducted, as most studies were published as government documents or independent research reports.

Two authors (SI and JR) developed the electronic search strategies, which included keywords from the conceptual domains of mortality, drugs and COVID-19 (detailed search strategies for all databases are available in Additional File 1). Two authors (SI and FN) executed the electronic search strategies, screened titles and abstracts and reviewed the full texts. Disagreements between the two authors in the selection of studies were resolved by consultation with a third author (JR). One author (SI) completed the data extraction, with subsequent review and verification by another author (FN). Multiple reports from included studies were considered in the data abstraction.

As the outcome definitions were expected to vary considerably between the included studies, findings were not pooled quantitatively in a meta-analysis, rather findings were synthesized qualitatively in a narrative synthesis. The findings were grouped and reported according to two comparison periods for drug overdose-related deaths after the onset of COVID-19: 1) months leading up to the pandemic in 2020 (e.g. March to April 2020 vs. January to February 2020); and 2) comparative months from 2019 (e.g. March to June 2020 vs. March to June 2019). Percentage changes in drug overdose-related deaths from these studies were summarized as range of effects and directionality of effects (through vote counting). Findings from all pertinent analyses were additionally presented, as available.

### Percentage change analyses

Data from national or subnational (state and provincial) government public health surveillance systems and reports were compiled through electronic searches of Google (using keywords such as “drug deaths”. “opioid deaths” and “overdose deaths”), review of a listing of opioid dashboards and data assembled by the Carolina Center for Health Informatics [20] and expert consultations (with membership of the Canadian Research Initiative on Substance Misuse). These electronic searches for the percentage change analyses were not systematic, although a similar set of keywords were utilized. To be eligible for data extraction and analysis, data had to be available according to or be amenable to formatting as annual quarters (Quarter 1 [Q1]: January to March; Q2: April to June; Q3: July to September; Q4: October to December) minimally from January 2020 to June 2020. No exclusions were made on the basis of other criteria, including current reporting status (preliminary, finalized).

Data pertaining to the broadest available category of drug overdose-related deaths were extracted until Q3 2020. For example, drug overdose-related deaths were extracted rather than opioid overdose-related deaths, fentanyl overdose-related deaths or psychostimulant overdose-related deaths, if data on all four were available. If such a broad category of drug overdose-related deaths was not available, data pertaining to opioid overdose-related deaths were extracted, as they represent the main driver of drug overdose-related deaths in most jurisdictions in North America. Subnational data were extracted for 12 jurisdictions in the United States (Connecticut, Indiana, Louisiana, Maine, Massachusetts, Mississippi, New Hampshire, New Jersey, Rhode Island, Vermont, Virginia, Washington) and national data were extracted for Canada. However, as the national data were available until Q2 2020 in Canada, additional subnational data that extended until Q3 2020 were extracted from Alberta, British Columbia and Quebec. The primary analysis included computation of percentage changes in drug overdose-related deaths comparing Q2 2020 with Q1 2020 separately for all jurisdictions. The secondary analyses included: 1) computation of analogous percentage changes comparing the subsequent quarters to characterize the trajectory of the impact of COVID-19; and 2) computation of analogous percentage changes comparing quarters from 2020 with quarters from 2019 to contextualize the impact of COVID-19.

### Ethics approval

All research activities were conducted as part of the Ontario Node of the Canadian Research Initiative on Substance Misuse Project. No additional review and approval was required, as aggregated data were extracted from publicly available studies and public health surveillance systems and reports.

## Results

### Systematic review

#### Characteristics of included studies

**Fig. 1:**
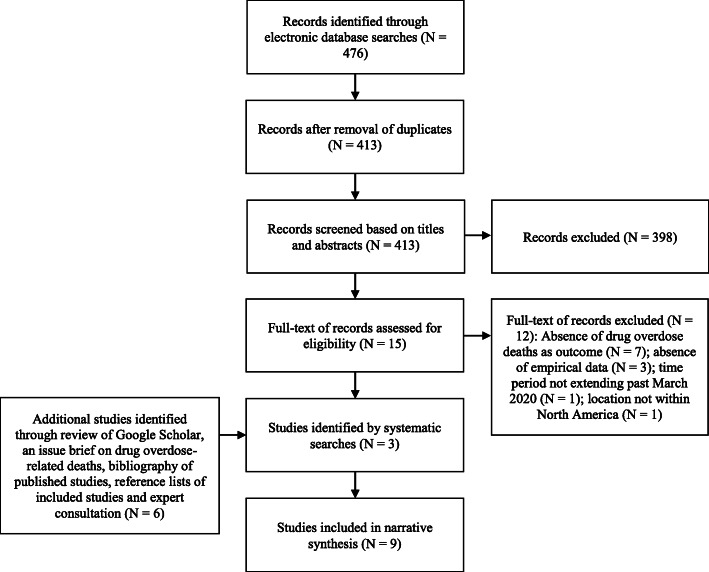
Preferred Reporting Items for Systematic Reviews and Meta-Analyses (PRISMA) Flow Diagram of Study Selection. The electronic database searches yielded 413 unique records. Based on title and abstract screening, 398 records were excluded and 15 records underwent full-text assessments. The additional searches yielded another six studies. After the application of the selection criteria, nine studies were included in the systematic review

After the application of the selection criteria, nine studies from 419 unique studies were included in the systematic review, all of them published in English (Fig. 1) [21–29]. Four included studies were published as government documents, three included studies were published as peer-reviewed manuscripts and two included studies were published as independent research reports.

The characteristics of all included studies are detailed in Table 1. Eight included studies were from the United States (Georgia, Kentucky, Michigan, Minnesota, Marion County [Indiana] and San Francisco [California]) and one included study was from Canada (Ontario). Although the maximum outcome assessment period in the included studies extended until September 2020, most included studies’ outcome assessment period did not extend past June 2020. The data sources for procuring the drug overdose-related deaths varied in the included studies, including emergency medical services systems, offices of the chief medical officer or coroner, vital records systems and other electronic surveillance systems. In a similar manner, the operationalization of drug overdose-related deaths in the included studies varied as well, with four of the nine included studies focusing exclusively on opioids.

**Table 1:** Characteristics of and Main Findings from the Studies Included in the Systematic Review

Study (Publication Type)	Location (Time Period)	Population	Outcome	Outcome Ascertainment and Definition	Main Findings
Centers for Disease Control and Prevention (2020) (Government Document)	United States of America (July 31, 2018 – May 31, 2020)	United States of America	Provisional drug overdose deaths, provisional synthetic opioid overdose deaths, provisional cocaine overdose deaths, provisional psychostimulant overdose deaths	Provisional drug overdose deaths were derived from death records received by the National Center for Health Statistics, which represent incomplete, underestimated counts derived from preliminary data that meet certain quality criteria and vary depending on the jurisdiction and 12-month ending period. Importantly, these counts were adjusted for incomplete reporting, and presented according to 12-month ending periods (counts of deaths occurring in the 12-month period ending in the indicated month). Provisional drug overdose deaths were defined as deaths with the ICD-10 Codes X40-X44, X60-X64, X85 and Y10-Y14 as underlying causes-of-death. Based on the involvement of other drug classes, provisional drug overdose deaths were further classified as provisional synthetic opioid overdose deaths (ICD-10: T40.4), provisional cocaine overdose deaths (ICD-10: 40.5) and provisional psychostimulant overdose deaths (ICD-10: T-43.6).	Approximately 81,230 provisional drug overdose deaths occurred in the United States in the 12-months ending in May 2020, which represented an 18% increase from the 12-months ending in June 2019. When examining by subnational jurisdictions, similar percentage changes in provisional drug overdose deaths amounted to more than 20% in 25 states and the District of Columbia, 10 to 19% in 11 states and New York City and 0 to 9% in 10 states. Increases per month in provisional drug overdose deaths were largest between the 12-months ending in March 2020 and the 12-months ending in April 2020 (2146 deaths) and between the 12-months ending in April 2020 and the 12-months ending in May 2020 (3388 deaths). The average increase per month in provisional drug overdose deaths amounted to 2348 deaths post COVID-19 (12-months ending in March 2020 to 12-months ending in May 2020) compared with 680 deaths pre COVID-19 (12-months ending in June 2019 to 12-months ending in February 2020). Compared with the 12-months ending in June 2019, provisional synthetic opioid overdose deaths were 38.4% higher in the 12-months ending in May 2020. When examining the subnational jurisdictions (*N* = 38), similar percentage changes amounted to more than 50% in 18 states, 25 to 49% in 11 states, 10 to 24% in 7 states and less than 1% in 1 state. The average increase per month in provisional synthetic opioid overdose deaths amounted to 2198 deaths post COVID-19 (12-months ending in March 2020 to 12-months ending in May 2020) compared with 770 deaths pre COVID-19 (12-months ending in June 2019 to 12-months ending in February 2020). Compared with the 12-months ending in June 2019, provisional cocaine overdose deaths were 26.5% higher and psychostimulant overdose deaths were 34.8% higher in the 12-months ending in May 2020. The average increase per month in provisional cocaine overdose deaths amounted to 653 deaths post COVID-19 (12-months ending in March 2020 to 12-months ending in May 2020) compared with 250 deaths pre COVID-19 (12-months ending in June 2019 to 12-months ending in February 2020). The average increase per month in provisional psychostimulant overdose deaths amounted to 758 deaths post COVID-19 (12-months ending in March 2020 to 12-months ending in May 2020) compared with 350 deaths pre COVID-19 (12-months ending in June 2019 to 12-months ending in February 2020).
Georgia Department of Public Health (2020) (Government Document)	Georgia, United States of America (December 2019 – June 2020)	State of Georgia	Drug-involved overdose deaths, opioid-involved overdose deaths, heroin-involved overdose deaths and fentanyl-involved overdose deaths	Drug-involved overdose deaths were based on death certificates from the Georgia Department of Public Health Vital Records, which included residents that died inside or outside of Georgia. Drug-involved overdose deaths were defined as those with ICD-10 Codes X40-X44, X60-X64, X85, Y10-Y14 as underlying causes-of-deaths (see [53] for additional details on the derivation of opioid-involved overdose deaths, heroin-involved overdose deaths and fentanyl-involved overdose deaths).	Drug-involved overdose deaths between March 15, 2020 and June 27, 2020 (444 deaths) were 9% higher compared with between December 1, 2019 and March 14, 2020 (484 deaths). A similar pattern of findings was evident for opioid-involved overdose deaths (25.3% [273 deaths to 342 deaths]), heroin-involved overdose deaths (32.3% [93 deaths to 123 deaths]) and fentanyl-involved overdose deaths (61.4% [140 deaths to 226 deaths]).
Giesel et al. (2020) (Government Document)	Minnesota, United States of America (January 2017 – June 2020)	State of Minnesota	Drug overdose deaths, opioid-involved drug overdose deaths, synthetic opioid-involved drug overdose deaths, prescription opioid-involved drug overdose deaths, heroin-involved drug overdose deaths, psychostimulant-involved drug overdose deaths, benzodiazepine-involved drug overdose deaths, cocaine-involved drug overdose deaths.	Drug overdose deaths were preliminarily derived from death certificates by the Injury and Violence Prevention Section in the Minnesota Department of Health.	Drug overdose deaths between April 2020 and June 2020 (277 deaths) were 30% higher compared with between January 2020 and March 2020 (213 deaths). The analogous percentage increase amounted to 13% between April 2019 and June 2019 (198 deaths) compared with between January 2019 and March 2019 (175 deaths), and 31% between January 2020 and June 2020 (490 deaths) compared with between January 2019 and June 2019 (373 deaths). Compared with between January 2019 and June 2019, between January 2020 and June 2020 opioid-involved drug overdose deaths increased 55% (from 197 deaths to 305 deaths), synthetic opioid-involved drug overdose deaths increased 74% (from 140 deaths to 244 deaths), prescription opioid-involved drug overdose deaths increased 56% (from 62 deaths to 97 deaths) and heroin-involved drug overdose deaths increased 52% (from 50 deaths to 76 deaths). Compared with between January 2019 and June 2019, between January 2020 and June 2020 psychostimulant-involved drug overdose deaths increased 55% (from 111 deaths to 172 deaths), benzodiazepine-involved drug overdose deaths increased 85% (from 39 deaths to 72 deaths) and cocaine-involved drug overdose deaths increased 67% (from 24 deaths to 40 deaths).
Glober et al. (2020) (Peer-Reviewed Manuscript)	Marion County, Indiana, United States of America (January 1, 2019 – July 7, 2020)	Marion County	Suspected accidental drug overdose deaths	Suspected accidental drug overdose deaths were sourced from the Marion County Coroner’s Office. In the event of pending toxicology results, circumstantial information (e.g. scene investigation), medical history and social history were used in the determination, as documented in the coroner’s reports.	Suspected accidental drug overdose deaths increased by 47% from 8.4 deaths per week in the 122-day period preceding March 25, 2020 to 12.4 deaths per week in the 105-day period following March 25, 2020 (*p* = 0.006). Suspected accidental drug overdose deaths increased by 104% from 6.1 deaths per week between March 25, 2019 and July, 24, 2019 to 12.4 deaths per week between March 25, 2020 and July 7, 2020 (p = < 0.001). Based on the time series from January 1, 2019 to March 24, 2020, autoregressive integrated moving average models further demonstrated that there was a several week period in between the stay-at-home order (March 25, 2020) and full state reopening when the suspected accidental drug overdose deaths per week were above the 99% confidence intervals of the forecast.
Ontario Drug Policy Research Network, Office of the Chief Coroner for Ontario/Ontario Forensic Pathology Service, Public Health Ontario, and Centre on Drug Policy Evaluation (2020) (Government Document)	Ontario, Canada (December 01, 2019 – June 30, 2020)	Province of Ontario	Opioid-related deaths	Opioid-related deaths were based on investigations of the Chief Coroner of Ontario and Ontario Forensic Pathology Service. These deaths were defined as acute intoxication or toxicity deaths that resulted from the direct contribution of an opioid (either alone or in conjunction with other substances). These deaths included both confirmed and suspected deaths (those with pending cause-of-death determination, but with evidence of drug involvement [i.e. evidence of drug use or drug paraphernalia at the scene and detection of opioids in post-mortem toxicology]).	Opioid-related deaths between March 16, 2020 and June 30, 2020 (695 deaths [46 deaths per week]) were 38.2% higher compared with between December 1, 2019 and March 15, 2020 (503 deaths [34 deaths per week]).
Overdose Mapping Application System (2020) (Independent Research Report)	United States of America (July 15, 2018 – May 06, 2020)	United States of America (Participating agencies from 48 states, District of Columbia and Puerto Rico)	Suspected fatal drug overdoses	Suspected fatal drug overdose deaths were entered in real-time to an electronic platform by first responders at the scene of incident, or pulled directly through record management systems (e.g. law enforcement) using an application program interface.	Rolling average of suspected fatal drug overdoses in the past 30 days increased by 11.39% from 2019 to 2020.
Rodda et al. (2020) (Peer-Reviewed Manuscript)	San Francisco, California, United States of America (January 01, 2020 – April 18, 2020)	City of San Francisco	Opioid overdose-related accidental deaths	Opioid overdose-related accidental deaths were sourced from the Office of the Chief Medical Examiner, which captured deaths with fentanyl, norfentanyl, 6-monoacetylmorphine, morphine and/or codeine detected in blood to determine their relevance in causation of drug toxicity.	Opioid overdose-related accidental deaths were higher between March 16, 2020 and April 18, 2020 (1.47 deaths per day [50 deaths in 34 days]) compared with between January 01, 2020 and March 15, 2020 (0.95 deaths per day [71 deaths in 75 days]). Opioid overdose-related accidental deaths per day were higher in January, February, March and April of 2020 compared with January, February, March and April of 2018 and 2019.
Slavova et al. (2020) (Peer- Reviewed Manuscript)	Kentucky, United States of America (January 14, 2020 – April 26, 2020)	State of Kentucky	Emergency medical services opioid overdose runs for suspected opioid overdose with death at the scene	Emergency medical services overdose runs for suspected opioid overdose with death at the scene were sourced from the Kentucky State Ambulance Reporting System.	Emergency medical services overdose runs for suspected opioid overdose with death at the scene between March 06, 2020 and April 26, 2020 (18 runs) were 50% higher compared with between January 14, 2020 and March 05, 2020 (12 runs).
System for Opioid Overdose Surveillance (2020) (Independent Research Report)	Michigan, United States of America (March 2019 – September 2020)	State of Michigan (12 counties with available data)	Suspected fatal opioid overdoses	Suspected fatal opioid overdoses were determined based on the electronic death database, Medicolegal Death Investigation Log (MDILog), as well as reports from county medical examiners. These deaths were determined by death investigators and updated daily to the System for Opioid Overdose Surveillance.	Suspected fatal opioid overdoses between March 1, 2020 and September 16, 2020 were 15% higher compared with suspected fatal opioid overdoses between March 1, 2019 and September 16, 2019.

The characteristics (including publication type, location, population, outcome and outcome ascertainment and definition) and main findings of the included studies are detailed. Eight included studies were from jurisdictions in the United States and one included study was from Canada, with the outcome assessment period in most included studies extending to June 2020. Data sources for procuring drug overdose-related deaths included emergency medical services systems, offices of the chief medical officer or coroner, vital records systems and other electronic surveillance systems. Four included studies focused exclusively on opioids. These studies underscored that drug overdose-related deaths after the onset of COVID-19 were higher compared with the months leading up to the pandemic in 2020 and comparative months in 2019

#### Narrative synthesis

The findings from all included studies are detailed in Table 1. Descriptive analyses (percentage changes and computations of deaths per day or month) represented the analytical strategies in all included studies, with autoregressive integrated moving average forecasting models additionally utilized in one included study. The findings are summarized hereunder for the broadest available category of drug overdose-related deaths after the onset of COVID-19 according to two comparison periods: 1) months leading up to the pandemic in 2020; and 2) comparative months in 2019.

In comparison to the months leading up to the pandemic in 2020, drug overdose-related deaths after the onset of COVID-19 were higher in seven studies [21–23, 26–29]. Drug overdose-related deaths were 9 to 50% higher between the periods of March 2020 to July 2020 compared with between the periods of December 2019 to March 2020 as observed in Georgia, Kentucky, Minnesota and Marion County [21, 23, 27, 28]. Opioid overdose-related deaths rose from 0.95 deaths per day between January 01, 2020 and March 15, 2020 to 1.47 deaths per day between March 16, 2020 and April 18, 2020 in San Francisco, representing a percentage increase of 55% [22]. Based on records received and processed at the time of analysis, drug overdose-related deaths in the 12-months ending in May 2020 were 18% higher compared with in the 12-months ending in June 2019 in the United States [29]. Importantly, the average increase per month in drug overdose-related deaths amounted to 2348 deaths after the onset of COVID-19 (12-months ending in March 2020 to 12-months ending in May 2020) compared with 680 deaths before the onset of COVID-19 (12-months ending in June 2019 to 12-months ending in February 2020) nationally in the United States [29]. Opioid overdose-related deaths in Ontario increased by 38% between March 16, 2020 and June 30, 2020 compared with between December 01, 2019 and March 15, 2020 [26].

In comparison with the comparative months in 2019, drug overdose-related deaths after the onset of COVID-19 were higher in five studies [22–25, 28]. Drug overdose-related deaths were 15, 40 and 104% higher between the periods of March 2020 to September 2020 compared with between the periods of March 2019 to September 2019, as observed in Michigan, Minnesota and Marion County, respectively [23, 24, 28]. Opioid overdose-related deaths were higher in January, February, March and April of 2020 compared with January, February, March and April of 2019 (6, 18, 15, 13 deaths per month vs. 27, 34, 35, 25 deaths per month, respectively) in San Francisco [22]. Based on agencies participating in the Overdose Detection Mapping Application Program System throughout the United States, rolling average of drug overdose-related deaths in the past 30 days were 11% higher between January and May 2020 compared with between January and December 2019 [25].

### Percentage change analysis

The findings from the percentage change analyses are presented in Table 2 (see Additional Files 2 and 3 for data sources, derivation notes and death counts). Jurisdictions in the United States reported variations of either drug overdose-related deaths or opioid overdose-related deaths. Drug overdose-related deaths increased by 2 to 60% in Q2 2020 compared with Q1 2020 in jurisdictions in the United States. Variations of either opioid overdose-related deaths or illicit drug overdose-related deaths were reported in jurisdictions in Canada. Drug overdose-related deaths increased 58% in Q2 2020 compared with Q1 2020 in Canada. During the same time period, drug overdose-related deaths more specifically increased 107% in Alberta, 80% in British Columbia and 28% in Quebec. Among jurisdictions that reported drug overdose-related deaths in Q3 2020, comparisons with Q2 2020 demonstrated increases in two jurisdictions in the United States and two jurisdictions in Canada (3 to 6% and 12 to 13%, respectively), and decreases in four jurisdictions in the United States and one jurisdiction in Canada (− 6% to − 30% and − 2%, respectively). On the other hand, drug overdose-related deaths in Q1 2020 compared with Q1 2019 demonstrated increases in all jurisdictions in the United States (1 to 72%), with the exception of Mississippi and New Hampshire. Decreases were evident in this regard in all examined jurisdictions in Canada (− 3% to − 9%). However, drug overdose-related deaths from Q1-Q2 2020 compared with Q1-Q2 2019 demonstrated increases in all but one jurisdiction in the United States and Canada (4 to 50%). This pattern of finding persisted when comparing Q1-Q3 2020 with Q1-Q3 2019 (2 to 62%).

**Table 2:** Percentage changes in drug overdose-related deaths in jurisdictions from North America

Jurisdiction	Outcome	Availability	Percentage Change (%) ^**a**^							
			2020			2020 vs. 2019				
			**Q2 vs. Q1**	**Q3 vs. Q1**	**Q3 vs. Q2**	**Q1**	**Q2**	**Q3**	**Q1-Q2**	**Q1-Q3**
**United States**										
Connecticut	Unintentional drug overdose deaths	Q1 2019–Q3 2020	25.0	18.0	−5.6	23.0	23.0	1.1	23.0	14.5
Indiana	Drug overdose deaths	Q1 2019–Q2 2020	20.6	–	–	14.3	36.2	–	25.3	–
Louisiana	Drug overdose deaths	Q1 2019 – Q2 2019; Q1 2020 – Q2 2020	32.6	–	–	26.9	62.2	–	44.9	–
Maine	Confirmed drug overdose death	Q1 2019–Q2 2020	3.1	–	–	71.6	28.4	–	46.6	–
Massachusetts	Opioid-related overdose deaths	Q1 2019–Q3 2020	17.8	−14.4	−27.3	1.4	19.0	−14.1	10.2	2.1
Mississippi	Suspected drug overdose deaths	Q1 2019–Q2 2020	60.0	–	–	−41.2	−18.6	–	−29.1	–
New Hampshire	Confirmed drug overdose deaths	Q1 2019–Q3 2020	4.7	−27.1	−30.4	−4.5	14.3	−15.2	4.3	−1.7
New Jersey	Suspected drug-related deaths	Q1 2019–Q3 2020	2.2	−8.1	−10.0	19.9	14.7	−11.5	17.2	6.4
Rhode Island	Accidental drug overdose deaths	Q1 2019–Q3 2020	5.3	11.7	6.1	22.1	25.3	41.9	23.7	29.6
Vermont	Opioid-related accidental and undetermined deaths	Q1 2019–Q3 2020	25.0	28.1	2.5	6.7	122.2	20.6	50.0	37.8
Virginia	Drug overdose deaths	Q1 2019–Q2 2020	40.3	–	–	13.3	66.8	–	39.4	–
Washington	Drug overdose deaths	Q1 2019–Q2 2020	12.1	–	–	17.7	37.2	–	27.3	–
**Canada**										
Canada	Apparent opioid toxicity deaths	Q1 2019–Q2 2020	58.2	–	–	−6.4	53.7	–	23.1	–
Alberta	Accidental acute opioid poisoning deaths	Q1 2019–Q3 2020	107.3	132.7	12.2	−7.4	65.4	132.7	31.7	62.0
British Columbia	Unintentional illicit drug toxicity deaths	Q1 2019–Q3 2020	79.6	75.5	−2.3	−8.5	94.8	114.5	38.7	60.6
Quebec	Suspected unintentional opioid or other drug poisoning deaths	Q1 2019–Q3 2020	27.6	44.0	12.8	−2.5	78.3	62.1	30.7	41.3

^a^ Q1: January – March; Q2: April – June; Q3: July – September; Q4: October – December

Percentage changes in drug overdose-related deaths in jurisdictions in the United States and Canada are displayed. Drug overdose-related deaths in Q2 2020 compared with Q1 2020 increased by 2 to 60% in jurisdictions in the United States and by 58% in Canada. Among jurisdictions that reported drug overdose-related deaths in Q3 2020, comparisons with Q2 2020 indicated that drug overdose-related deaths increased in two jurisdictions in the United States and two jurisdictions in Canada, and decreased in four jurisdictions in the United States and one jurisdiction in Canada. Drug overdose-related deaths in Q1 2020 compared with Q1 2019 increased in most jurisdictions in the United States and decreased in all jurisdictions in Canada. Drug overdose-related deaths in Q1-Q2 2020 compared with Q1-Q2 2019 and Q1-Q3 2020 compared with Q1-Q3 2019 increased in most jurisdictions in the United States and Canada

## Discussion

### Summary of findings

Observational studies that examined the impact of COVID-19 on drug overdose-related deaths in the United States and Canada were reviewed. Drug overdose-related deaths after the onset of COVID-19 were higher compared with the months leading up to the pandemic in 2020 or comparative months in 2019. In additional percentage change analyses based on data from public health surveillance systems and reports, drug overdose-related deaths increased by 2 to 60% in jurisdictions in the United States and by 58% in Canada between Q1 2020 and Q2 2020. These findings indicate that drug overdose-related deaths increased after the onset of COVID-19.

### Comparison with previous studies

Two rapid reviews have examined the impact of COVID-19 on drug overdose-related deaths based on empirical evidence [13, 14]. As they were both conducted during the early stages of COVID-19, there is minimal overlap with the studies or the public health surveillance systems data included hereunder [13, 14]. Largely on the basis of public health surveillance systems in Canada, increases in drug overdose-related deaths in British Columbia and Ontario (including Toronto and Simcoe Muskoka District Health Unit) were highlighted [13, 14]. However, it was difficult to conclude whether the increases were attributable to the toxic drug supply, COVID-19 or a combination of both [13, 14]. In a subsequent assessment from Philadelphia that was published after the completion of the systematic searches, Khatri and colleagues demonstrated increases in opioid overdose-related deaths between April 2020 and June 2020 compared with between December 2019 and February 2020 among non-Hispanic Blacks (mean monthly overdose deaths of 49 deaths vs. 30 deaths; *p* = 0.04), but not among non-Hispanic Whites (35 deaths vs. 45 deaths; *p* = 0.11) or Hispanics (14 deaths vs. 13 deaths; *p* = 0.57) [30].

### Interpretation and implications

Drug overdose-related deaths increased prior to and accelerated after the onset of COVID-19 in the United States. In contrast, drug overdose-related deaths stabilized prior to the onset of COVID-19, but increased after the onset of COVID-19 in Canada. Although seasonal variations (e.g. due to weather and holidays) may have contributed to some extent to the increase in drug overdose-related deaths, they probably do not explain the entirety of the findings. The percentage change in drug overdose-related deaths between Q1 and Q2 was 3% (mean; range − 40 to 38%) in 2019 and 21% (mean; range 2 to 60%) in 2020 in jurisdictions in the United States and − 4% in 2019 and 58% in 2020 in Canada, which suggests the potential role of the consequences of COVID-19. However, given the absence of studies with stronger methodological designs (e.g. interrupted time series design and regression discontinuity design), it is difficult to draw causal inferences because the impact of other factors cannot be ruled out. As such, these conclusions are preliminary that require further confirmation in future research.

Despite the preliminary nature of the current state of the evidence, it is clear that the rise in drug overdose-related deaths after the onset of COVID-19 necessitates actions on five fronts. The applicability of these recommendations will vary depending on local jurisdiction laws. First, expanded access to substance use disorder treatment needs to be facilitated, especially the delivery of services through innovative models of care to ensure minimal disruptions in treatment. Notably, telemedicine has facilitated low-barrier access to opioid pharmacotherapy with buprenorphine in the United States, as the federal regulatory changes enacted during the pandemic now permit clinicians to prescribe buprenorphine after conducting an initial visit using audio-visual or telephonic assessments rather than in-person assessments [31]. Removal of the in-person assessment requirement has resulted in rapid initiation of treatment, often including same day assessments, prescriptions and inductions [31, 32]. In addition, given the recent rise in psychostimulant overdose-related deaths [33, 34], treatment options for both opioid use disorder and psychostimulant use disorder are needed. Second, undisrupted access to harm reduction services needs to be ensured, including drug checking programs that make use of a wide range of technologies (e.g. fourier-transform infrared (FTIR) spectrometers, fentanyl test strips and drug testing kits), needle and syringe exchange programs and supervised consumption services, all of which have demonstrated varied range of benefits in terms of injecting risk behaviors, bloodborne infectious diseases and drug overdoses [35–37]. Third, drug overdose prevention needs to emphasize risk reduction strategies during episodes of drug consumption: not to use drugs alone, determine the contents of drugs beforehand, ensure availability of naloxone on-hand and have someone check-in during consumption [29]. In particular, novel strategies such as virtual drug use spotting through social networks (e.g. phone calls, video calls) or technological innovations (e.g. smart phone applications) can be drawn upon when using drugs alone, an intervention where substances are consumed under observation and emergency services are alerted in the event of unresponsiveness [38, 39]. Wider availability of naloxone is similarly imperative in the current context, which has been shown to increase drug overdose emergency visits and decrease drug overdose deaths [40]. Fourth, to reduce drug use attributable risks and harms due to the toxic drug supply in North America, access needs to be provided to a safe drug supply (defined as legal and regulated supply of psychoactive substances that are typically available from the illegal drug market) [41, 42]. Fifth, the unprecedented circumstances suggest that there is a need for decriminalization of drug use, which will prioritize health over punishment, reduce stigma surrounding drug use and enable earlier engagement with prevention and treatment interventions [41, 43, 44]. Importantly, although these recommendations were relevant prior to the onset of COVID-19, their expedited, large-scale adoption is now high-priority given the rise in drug overdose-related deaths. In addition to these specific recommendations to address drug overdose-related deaths, prioritized access to vaccinations needs to be ensured for PWUD [45], who have intersecting social and health vulnerabilities that may elevate the risk for the infection, complications and mortality due to COVID-19 [46–48]. PWUD experience homelessness and incarceration, high prevalence of pre-existing chronic health conditions, inadequate access to shelters and barriers to harm reduction services [8, 10, 49–51]. In addition, substance use characteristics such as sharing paraphernalia and inability to social distance or self-isolate may further amplify the risks due to COVID-19 [49]. As such, access to vaccination is a priority for this population, which can be facilitated through the existing physical infrastructure of harm reduction and addiction treatment services [45].

### Limitations

There are some limitations that should be considered in the interpretation of the findings. First, non-fatal drug overdoses were not considered in the systematic review or percentage change analyses. As such, the full extent of the impact of COVID-19 on drug overdoses is probably larger. Second, despite the broad search strategies and expert consultations, studies from Canada were not well represented and studies from Mexico were not found in the systematic review, limiting the generalizability of the findings. In a similar manner, as the percentage change analyses included 12 jurisdictions from its states and territories, the findings may not be generalizable to the rest of the United States. Third, given the heterogenous definitions of drug overdose-related deaths in the included studies in the systematic review, quantitative synthesis of the findings through meta-analysis was not possible. Fourth, due to the need for timely evidence in the context of a rapidly escalating health crisis, data in some of the included studies in the systematic review were preliminary and subject to change, based on health surveillance systems or health administrative databases that are susceptible to misclassification bias and lacked confirmation through toxicological analyses or physician diagnoses. On that same note, data from all jurisdictions in the percentage change analyses were preliminary and subject to change. The onset of COVID-19 in particular has underscored the urgent need for standardized, real-time statistics on drug overdose-related deaths, as they are not available consistently across jurisdictions in North America. These limitations concerning the outcome assessment may have impacted the validity of the findings. Fifth, outcome assessment period in the included studies in the systematic review or percentage change analyses did not extend past September 2020, warranting a need for continued monitoring of drug overdose-related deaths. Sixth, stronger methodological designs were absent from most studies, and descriptive analyses (without statistical tests of differences) represented the analytical strategy in all but one of the included studies in the systematic review. Percentage change analyses were operationalized hereunder, as the granular data required for interventional time series analyses were not publicly available.

## Conclusion

Drug overdose-related deaths after the onset of COVID-19 were higher compared with the months leading up to the pandemic in 2020 or comparative months in 2019 in the United States and Canada. Similar increases were reflected in percentage change analyses of drug overdose-related deaths comparing Q2 2020 with Q1 2020. Although these findings are widespread, further confirmation is needed, as the impact of other factors cannot be ruled out, given the ecological nature of the data [52]. The current situation necessitates a multi-pronged approach, encompassing expanded access to substance use disorder treatment, undisrupted access to harm reduction services, emphasis on risk reduction strategies, provision of a safe drug supply and decriminalization of drug use.

## Supplementary Information

**Additional File 1.** Provides further details on the methodology and additional tables and figures to support the results pertaining to the systematic review reported in the main text of the manuscript

**Additional File 2.** Provides further details on the data to support the results pertaining to the percentage change analyses reported in the main text of the manuscript

**Additional File 3.** Provides data to support the results pertaining to the percentage change analyses reported in the main text of the manuscript

## Data Availability

All data generated or analysed during this study are included in this article and the additional files.

## Abbreviations

COVID-19: Novel Coronavirus Disease
PRISMA: Preferred Reporting Items for Systematic Reviews and Meta-Analyses
PWUD: People Who Use Drugs
Q: Annual Quarter
SARS-CoV-2: Severe Acute Respiratory Syndrome Coronavirus
