# Supplementary material for: The impact of the novel coronavirus disease (COVID-19) pandemic on drug overdose-related deaths in the United States and Canada: a systematic review of observational studies and analysis of public health surveillance data
Source: Subst Abuse Treat Prev Policy. 2021 Nov 29;16:87. doi: 10.1186/s13011-021-00423-5 (PMC8628272; doi:10.1186/s13011-021-00423-5)
Supplement: Supplementary file 2 — Additional File 2. Provides further details on the data to support the results pertaining to the percentage change analyses reported in the main text of the manuscript [file 13011_2021_423_MOESM2_ESM.docx]

**Additional File 2:** The impact of the novel coronavirus disease (COVID-19) pandemic on drug overdose-related deaths in the United States and Canada: A systematic review of observational studies and analysis of public health surveillance data

This document provides further details on the data to support the results pertaining to the percentage change analyses reported in the main text of the manuscript.

| Additional Details About the Data | Page 2 |
| --- | --- |

**Additional Details About the Data**

**CONNECTICUT (UNITED STATES)**

**Source:** https://portal.ct.gov/-/media/DPH/Injury-Prevention/Opioid-Overdose-Data/November-2020-and-2019-Drug-Overdose-Deaths-Monthly-Report_Updated-_12-10-2020.pdf

**Date of Extraction:** December 16/2020

**Outcome (Availability):** Unintentional drug overdose deaths (Q1 2019 - Q3 2020)

**Data Notes:** 1) Data are subject to change for 2020; 2) Data includes confirmed cases of fatal drug overdose; 3) Data are sourced from the Connecticut Office of the Chief Medical Examiner

**INDIANA (UNITED STATES)**

**Source:** https://www.in.gov/isdh/27393.htm

**Date of Extraction:** December 16/2020

**Outcome (Availability):** Drug overdose deaths (Q1 2019 – Q2 2020)

**Data Notes:** 1) Data are provisional for 2020; 2) Data exclude out of state deaths of Indiana residents; 3) Data are based on death certificate information recorded by county coroner or medical examiners; 4) Drug overdose deaths are defined as drug poisoning deaths (ICD-10 Codes: X40-X44, X60-X64, X85, Y10-Y14) with specific drug categories as contributing causes (ICD-10: T40.0, T40.1, T40.2, T40.3, T40.4, T40.5, T40.6, T42.4, T43.6).

**LOUISIANA (UNITED STATES)**

**Source:** https://ldh.la.gov/index.cfm/page/3192

**Date of Extraction:** December 15/2020

**Outcome (Availability):** Drug overdose deaths (Q1 2019 - Q2 2019; Q1 2020 - Q2 2020)

**Data Notes:** 1) Data are preliminary and subject to change for 2020; 2) Definition of drug deaths is not indicated.

**MAINE (UNITED STATES)**

**Source:** <https://www.maine.gov/dhhs/mecdc/infectious-disease/epi/syndromic/> and <http://www.maine.gov/tools/whatsnew/attach.php?id=3484620&an=1>

**Date of Extraction:** December 16/2020

**Outcome (Availability):** Confirmed drug overdose deaths (Q1 2019 – Q2 2020)

**Data Notes:** 1) Data are sourced from the Office of the Attorney General's Office of the Chief Medical Examiner; 2) Confirmed drug overdose death is defined as a death with mention of one or more drugs on the death certificate as a cause or significant contributing factor; 3) Confirmed drug overdose deaths include all manner of death (intentional and accidental).

**MASSACHUSETTS (UNITED STATES)**

**Source:** https://www.mass.gov/doc/opioid-related-overdose-deaths-among-ma-residents-november-2020/download

**Date of Extraction:** December 16/2020

**Outcome (Availability):** Opioid-related overdose deaths (Q1 2019 - Q3 2020)

**Data Notes:** 1) Data are preliminary and subject to change; 2) Data includes opioid-related overdose deaths from all intents, including unintentional, undetermined or suicide deaths; 3) Opioids include heroin, illicitly manufactured fentanyl, opioid-based prescription painkillers, and other unspecified opioids; 4) Opioid-related overdose deaths were defined as deaths with poisonings or overdoses as underlying causes of death (ICD-10 Codes: X40-X44, X60-X64, X85, and Y10-Y14), with opioids coded as a multiple cause of death (ICD-10 Code: T40.0, T40.1, T40.2, T40.3, T40.4, and T40.6); 5) Opioid-related overdose deaths include probable opioid-related overdose deaths. These deaths are based on predictive modeling techniques for cases not yet finalized by the Office of the Chief Medical Examiner. Contributing to the predictive model are various data from the Office of the Chief Medical Examiner and the Massachusetts State Police. The model includes information from the death certificate, Medical Examiner’s notes, and the determination by the State Police of a suspected heroin death.

**MISSISSIPPI (UNITED STATES)**

**Source:** <https://msdh.ms.gov/msdhsite/_static/resources/11184.pdf> and https://msdh.ms.gov/msdhsite/_static/resources/10485.pdf

**Date of Extraction:** December 16/2020

**Outcome (Availability):** Suspected drug overdose deaths (Q1 2019 – Q2 2020)

**Data Notes:** 1) Data are preliminary; 2) Data do not include those deaths with pending toxicology reports; 3) Data reflect when the coroners received the reports, regardless of when the deaths occurred; 4) Data maybe incomplete, as not all coroners report on suspected drug overdose deaths; 5) Suspected drug overdose deaths are based on reports filed by the coroners.

**NEW HAMPSHIRE (UNITED STATES)**

**Source:** <https://www.dhhs.nh.gov/dcbcs/bdas/documents/dmi-2020-overview.pdf> and https://www.dhhs.nh.gov/dcbcs/bdas/documents/2019-dmi-overview.pdf

**Date of Extraction:** December 15/2020

**Outcome (Availability):** Confirmed drug overdose deaths (Q1 2019 - Q3 2020)

**Data Notes:** 1) Data are finalized for 2019 but not for 2020; 2) Data are sourced from the New Hampshire Medical Examiner's Office**;** 3) Confirmed drug overdose deaths represent confirmed deaths.

**NEW JERSEY (UNITED STATES)**

**Source:** https://www.state.nj.us/health/populationhealth/opioid/

**Date of Extraction:** December 15/2020

**Outcome (Availability):** Suspected drug-related deaths (Q1 2019 - Q3 2020)

**Data Notes:** 1) Data are preliminary and subject to change; 2) Data are obtained from New Jersey Office of the State Medical Examiner; 3) Suspected drug-related deaths are based on the decedent's history, death scene investigation, autopsy report and toxicology results.

**RHODE ISLAND (UNITED STATES)**

**Source:** https://health.ri.gov/data/drugoverdoses/

**Date of Extraction:** December 15/2020

**Outcome (Availability):** Accidental drug-related overdose deaths (Q1 2019 - Q3 2020)

**Data Notes:** 1) Data are not complete and do not reflect the total number of deaths for September 2020; 2) Accidental drug-related overdose deaths included deaths in Rhode Island that were that were accidental in nature; had a drug listed on the drug certificate, which caused the death or was a contributing factor to the death; and received confirmation from the Chief Medical Examiner.

**VERMONT (UNITED STATES)**

**Source:** <https://www.healthvermont.gov/sites/default/files/documents/pdf/ADAPMonthlyOpioidRelatedFatalities.pdf> and https://www.healthvermont.gov/sites/default/files/documents/pdf/ADAP_Data_Brief_Opioid_Related_Fatalities.pdf

**Date of Extraction:** December 15/2020

**Outcome (Availability):** Opioid-related accidental and undetermined deaths (Q1 2019 - Q3 2020)

**Notes:** 1) Data are preliminary for 2019 and 2020; 2) Data are sourced from the Vermont Department of Health Vital Statistics System; 3) Data include Vermont residents that died inside of Vermont and outside of Vermont; 4) Opioid-related accidental and undetermined deaths are defined as deaths that were accidental or undetermined in nature, with all of them involving at least one illicit or prescription opioid (for derivation methodology see https://www.healthvermont.gov/sites/default/files/documents/pdf/ADAP_Data_Brief_Opioid_Related_Fatalities.pdf).

**VIRGINIA (UNITED STATES)**

**Source:** https://www.vdh.virginia.gov/medical-examiner/forensic-epidemiology/

**Date of Extraction:** December 15/2020

**Outcome (Availability):** Drug overdose deaths (Q1 2019 – Q2 2020)

**Data Notes:** 1) Data are preliminary and subject to change; 2) Data are sourced from Virginia Medical Examiner Database System, which contains detailed information reported to the Office of the Chief Medical Examiner; 3) Data are based on accepted cases of either full autopsy or external exams; 4) Data are based on the locality of the death rather than the residence of the decedent; 5) Drug overdose deaths include those deaths where drugs caused or contributed to death, and excludes those deaths where drugs were detected but did not contribute to or caused death. All manners of death are included (i.e. accident, homicide, suicide and undetermined).

**WASHINGTON (UNITED STATES)**

**Source:** https://www.doh.wa.gov/Portals/1/Documents/8300/wa_lhj_quarterly_report_18_1_2_pub.html#3_README_Notes

**Date of Extraction:** December 15/2020

**Outcome (Availability):** Drug overdose death (Q1 2019 – Q2 2020)

**Data Notes:** 1) Data are preliminary for 2019 and 2020; 2) Data represent deaths by geography of the death of occurrence; 3) Drug overdose deaths are those with the ICD-10 Codes X40-X44, X60-X64, X85, Y10-Y14 as underlying causes-of-deaths.

**CANADA**

**Source:** https://health-infobase.canada.ca/substance-related-harms/opioids-stimulants

**Date of Extraction:** December 22/2020

**Outcome (Availability):** Apparent opioid toxicity deaths (Q1 2019 - Q2 2020)

**Data Notes:** 1) Data are preliminary and subject to change; 2) Data from Manitoba were not included from October 2019 to June 2020, and data from British Columbia encompassed illicit drug toxicity deaths rather than opioid toxicity deaths (for further differences in provincial reporting see <https://health-infobase.canada.ca/substance-related-harms/opioids-stimulants/technical-notes>); 3) Data included all types of investigations (completed or ongoing) and all manners (accident, suicide or unintentional); 4) Apparent opioid toxicity deaths are defined as substance use attributable intoxication or poisoning deaths that involve an opioid regardless of its source of procurement (i.e. illegal or prescription).

**ALBERTA (CANADA)**

**Source:** https://open.alberta.ca/dataset/f4b74c38-88cb-41ed-aa6f-32db93c7c391/resource/e8c44bab-900a-4af4-905a-8b3ef84ebe5f/download/health-alberta-covid-19-opioid-response-surveillance-report-2020-q2.pdf

**Date of Extraction:** December 22/2020

**Outcome (Availability):** Accidental acute opioid poisoning deaths (Q1 2019 – Q3 2020)

**Data Notes:** 1) Recent data may be less complete due to delays in data submission. As such, the data are subject to change; 2) Accidental acute opioid poisoning deaths have been certified by the Medical Examiner, indicating that all available evidence has been examined to list the cause of death on the death certificate. However, such deaths in the context of fentanyl are determined by presence of fentanyl in toxicology and initial circumstances suggesting a drug poisoning death; 3) Accidental acute opioid poisoning deaths included those deaths where preliminary evidence suggested the death was most likely due to opioid overdose.

**BRITISH COLUMBIA (CANADA)**

**Source:** https://www2.gov.bc.ca/assets/gov/birth-adoption-death-marriage-and-divorce/deaths/coroners-service/statistical/illicit-drug.pdf

**Date of Extraction:** December 16/2020

**Outcome (Availability):** Unintentional illicit drug toxicity deaths (Q1 2019 – Q3 2020)

**Data Notes:** 1) More recent data are based on preliminary circumstances. As such, it is subject to change as investigations are concluded; 2) Unintentional illicit drug toxicity deaths included accidental and undetermined deaths that involved illicit drugs (e.g. heroin, cocaine, methamphetamine, illicit fentanyl, MDMA) and unprescribed medications, as well as combinations of illicit drugs and unprescribed medications with other prescribed medications.

**QUEBEC (CANADA)**

**Source:** https://www.inspq.qc.ca/surdoses-opioides/deces-attribuables-une-intoxication-aux-opioides/deces-relies-une-intoxication-suspectee-aux-opioides-ou-autres-drogues-au-quebec-2017-2020

**Date of Extraction:** December 22/2020

**Outcome (Availability):** Suspected unintentional opioid or other drug poisoning (Q1 2019 – Q3 2020)

**Data Notes:** 1) Data are sourced from the Coroner’s Office; 2) Data are preliminary and subject to change, as the causes of death are still under investigation.
