# Supplementary material for: The impact of the novel coronavirus disease (COVID-19) pandemic on drug overdose-related deaths in the United States and Canada: a systematic review of observational studies and analysis of public health surveillance data
Source: Subst Abuse Treat Prev Policy. 2021 Nov 29;16:87. doi: 10.1186/s13011-021-00423-5 (PMC8628272; doi:10.1186/s13011-021-00423-5)
Supplement: Supplementary file 1 — Additional File 1. Provides further details on the methodology and additional tables and figures to support the results pertaining to the systematic review reported in the main text of the manuscript [file 13011_2021_423_MOESM1_ESM.docx]

**Additional File 1:** The impact of the novel coronavirus disease (COVID-19) pandemic on drug overdose-related deaths in the United States and Canada: A systematic review of observational studies and analysis of public health surveillance data

This document provides further details on the methodology and additional tables and figures to support the results pertaining to the systematic review reported in the main text of the manuscript.

| Additional Details on the Methodology | Page 2 |
| --- | --- |
| Web Tables | Page 8 |

**Additional Details on the Methodology**

**Electronic Search Strategies**

The electronic search strategies operationalized in each of the databases are presented in Table S1.

**Table S1: Search strategies operationalized in Medline, Embase, PsycInfo and Google Scholar**

| Medline | 1. covid-19.mp. 2. SARS-CoV-2.mp. 3. 2019-nCoV.mp. 4. exp Coronaviridae/ or Coronaviridae.mp. 5. Coronavirus infection.mp. or exp Coronavirus Infections/ 6. exp Coronavirus/ or Coronavirus.mp. 7. Pneumonia, Viral.mp. or exp Pneumonia, Viral/ 8. exp Betacoronavirus/ or Betacoronavirus.mp. 9. SARS Virus.mp. or exp SARS Virus/ 10. Severe Acute Respiratory Syndrome.mp. or exp Severe Acute Respiratory Syndrome/ 11. 1 or 2 or 3 or 4 or 5 or 6 or 7 or 8 or 9 or 10 12. Mortal$.mp. 13. exp Mortality/ 14. fatal$.mp. or exp Fatal Outcome/ 15. exp Death, Sudden/ or death$.mp. or exp "Cause of Death"/ or exp Death/ 16. 12 or 13 or 14 or 15 17. exp Heroin Dependence/ or exp Heroin/ or heroin.mp. 18. opiate.mp. or exp Opiate Alkaloids/ 19. opium.mp. or exp Opium Dependence/ or exp Opium/ or exp Opioid-Related Disorders/ or exp Substance-Related Disorders/ or exp Analgesics, Opioid/ 20. opioid.mp. 21. exp Morphine Dependence/ or morphine.mp. or exp Morphine/ 22. narcotics.mp. or exp Narcotics/ 23. Cocaine.mp. or exp Cocaine-Related Disorders/ or exp Cocaine/ or exp Crack Cocaine/ or exp Cocaine Smoking/ 24. exp Amphetamine-Related Disorders/ or exp Amphetamine/ 25. exp Amphetamines/ 26. exp Dextroamphetamine/ 27. exp Methamphetamine/ 28. exp Benzphetamine/ 29. exp N-Methyl-3,4-methylenedioxyamphetamine/ 30. exp Lisdexamfetamine Dimesylate/ 31. exp Methylphenidate/ 32. exp Phentermine/ 33. exp Chlorphentermine/ 34. exp Mephentermine/ 35. exp Iofetamine/ 36. exp p-Hydroxyamphetamine/ 37. exp 2,5-Dimethoxy-4-Methylamphetamine/ 38. exp p-Chloroamphetamine/ 39. (ATS or amphet* or meth?amphet* or deoxyephedrine or desoxy* or madrine or metamfet* or methylamphet* or n?methylamphet* or d?amphet* or dextro?amphet* or dexamphet* or dexedrine or stimulant* or ecstasy or MDMA or lisdexamfetamine or methylphenidate or modafinil).mp. [mp=title, abstract, original title, name of substance word, subject heading word, floating sub-heading word, keyword heading word, organism supplementary concept word, protocol supplementary concept word, rare disease supplementary concept word, unique identifier, synonyms] 40. illicit drugs.mp. or exp Illicit Drugs/ 41. 17 or 18 or 19 or 20 or 21 or 22 or 23 or 24 or 25 or 26 or 27 or 28 or 29 or 30 or 31 or 32 or 33 or 34 or 35 or 36 or 37 or 38 or 39 or 40 42. 11 and 16 and 41 |
| --- | --- |
| Embase | 1. exp death/ or Mortal*.mp. or exp mortality/ 2. fatal*.mp. or exp fatality/ 3. death*.mp. or exp "cause of death"/ or exp accidental death/ or exp sudden death/ 4. 1 or 2 or 3 5. cocaine.mp. or exp cocaine/ or exp cocaine smoking/ or exp cocaine dependence/ or exp cocaine derivative/ 6. heroin.mp. or exp diamorphine/ 7. opiate.mp. or exp opiate addiction/ or exp opiate/ 8. exp heroin dependence/ 9. opioid.mp. 10. opium.mp. 11. exp morphine addiction/ or exp morphine/ or morphine.mp. 12. (ATS or amphet* or meth?amphet* or deoxyephedrine or desoxy* or madrine or metamfet* or methylamphet* or n?methylamphet* or d?amphet* or dextro?amphet* or dexamphet* or dexedrine or stimulant* or ecstasy or MDMA or lisdexamfetamine or methylphenidate or modafinil).mp. [mp=title, abstract, original title, name of substance word, subject heading word, floating sub-heading word, keyword heading word, organism supplementary concept word, protocol supplementary concept word, rare disease supplementary concept word, unique identifier, synonyms] 13. exp amphetamine abuse/ or exp amphetamine/ or exp amphetamine derivative/ or exp amphetamine dependence/ 14. exp methamphetamine dependence/ or exp methamphetamine/ or exp methamphetamine-induced psychosis/ 15. exp dexamphetamine/ 16. exp iofetamine i 123/ or exp iofetamine/ or exp iofetamine i 125/ 17. exp 4 methoxyamphetamine/ 18. exp hydroxyamphetamine/ 19. exp mephentermine/ 20. exp phentermine/ 21. exp benzphetamine/ 22. exp chloramphetamine/ 23. exp chlorphentermine/ 24. exp midomafetamine/ 25. exp methylphenidate/ 26. exp lisdexamfetamine/ 27. exp modafinil/ 28. illegal drugs.mp. or exp illicit drug/ 29. exp addiction/ or exp substance abuse/ or exp drug dependence/ 30. 5 or 6 or 7 or 8 or 9 or 10 or 11 or 12 or 13 or 14 or 15 or 16 or 17 or 18 or 19 or 20 or 21 or 22 or 23 or 24 or 25 or 26 or 27 or 28 or 29 31. exp Coronavirinae/ or covid-19.mp. 32. coronavirus.mp. 33. Coronavirinae.mp. 34. Coronavirus infection.mp. or exp severe acute respiratory syndrome/ or exp Coronavirus infection/ or exp SARS coronavirus/ or exp Coronavirus/ 35. Pneumonia, Viral.mp. or exp virus pneumonia/ 36. SARS Virus.mp. 37. Severe Acute Respiratory Syndrome.mp. 38. Betacoronavirus.mp. or exp Betacoronavirus/ 39. SARS-CoV-2.mp. 40. 2019-nCoV.mp. 41. 31 or 32 or 33 or 34 or 35 or 36 or 37 or 38 or 39 or 40 42. 4 and 30 and 41 |
| PsycInfo | 1. exp "Death and Dying"/ or Mortal*.mp. 2. fatal*.mp. 3. exp Sudden Death/ or death*.mp. 4. exp Mortality Rate/ 5. exp mortality/ 6. 1 or 2 or 3 or 4 or 5 7. (ATS or amphet* or meth?amphet* or deoxyephedrine or desoxy* or madrine or metamfet* or methylamphet* or n?methylamphet* or d?amphet* or dextro?amphet* or dexamphet* or dexedrine or stimulant* or ecstasy or MDMA or lisdexamfetamine or methylphenidate or modafinil).mp. [mp=title, abstract, original title, name of substance word, subject heading word, floating sub-heading word, keyword heading word, organism supplementary concept word, protocol supplementary concept word, rare disease supplementary concept word, unique identifier, synonyms] 8. exp Dextroamphetamine/ 9. exp Methamphetamine/ 10. exp Amphetamine/ 11. exp Dextroamphetamine/ or exp Methylenedioxymethamphetamine/ 12. exp Methylphenidate/ 13. exp Crack Cocaine/ or exp Cocaine/ or cocaine.mp. 14. exp Heroin Addiction/ or exp Heroin/ or Heroin.mp. 15. exp Opiates/ or opiate.mp. 16. exp Drug Addiction/ or exp Drug Usage/ or exp Drug Dependency/ or exp "Substance Use Treatment"/ or exp Drug Abuse/ or exp Morphine/ or opium.mp. 17. exp Morphine Dependence/ or exp Morphine/ or morphine.mp. 18. opioid.mp. 19. exp Drug Addiction/ or exp Drug Usage/ or exp Drug Abuse/ or exp Drugs/ or exp "Substance Use Treatment"/ or illegal drug.mp. 20. 7 or 8 or 9 or 10 or 11 or 12 or 13 or 14 or 15 or 16 or 17 or 18 or 19 21. covid-19.mp. or exp Coronavirus/ 22. SARS-CoV-2.mp. 23. 2019-nCoV.mp. 24. Coronavirus.mp. 25. exp Severe Acute Respiratory Syndrome/ or Severe Acute Respiratory Syndrome.mp. 26. SARS Virus.mp. 27. Coronavirus Infection.mp. 28. betacoronavirus.mp. 29. coronaviridae.mp. 30. pneumonia.mp. or exp Pneumonia/ 31. 21 or 22 or 23 or 24 or 25 or 26 or 27 or 28 or 29 or 30 32. 6 and 20 and 31 |
| Google Scholar | (COVID-19 OR SARS-CoV-2 OR 2019-nCoV OR Coronavirus) AND (Mortal* OR Fatal* OR Death*) AND (Heroin OR Opiate OR Opium OR Opioid OR Morphine OR Narcotic* OR Cocaine OR Crack OR Amphet* OR Meth* OR Stimulant* OR Ecstasy OR MDMA OR Overdose* OR Toxicit*) |

**Web Tables**

**Table S1: Preferred Reporting Items for Systematic Reviews and Meta-Analyses Checklist**

| **Topic** | **Item Number** | **Checklist Item** | **Reported In** |
| --- | --- | --- | --- |
|  |  |  |  |
| **Title** |  |  |  |
| Title | 1 | Identify the report as a systematic review, meta-analysis, or both. | Title Page |
|  |  |  |  |
| **Abstract** |  |  |  |
| Structured summary | 2 | Provide a structured summary including, as applicable: background; objectives; data sources; study eligibility criteria, participants, and interventions; study appraisal and synthesis methods; results; limitations; conclusions and implications of key findings; systematic review registration number. | Abstract |
|  |  |  |  |
| **Introduction** |  |  |  |
| Rationale | 3 | Describe the rationale for the review in the context of what is already known. | Introduction (Paragraphs 1 to 3) |
| Objectives | 4 | Provide an explicit statement of questions being addressed with reference to participants, interventions, comparisons, outcomes, and study design (PICOS). | Introduction (Paragraph 3) |
|  |  |  |  |
| **Methods** |  |  |  |
| Protocol and registration | 5 | Indicate if a review protocol exists, if and where it can be accessed (e.g., Web address), and, if available, provide registration information including registration number. | Methods (Systematic Review: Paragraph 1) |
| Eligibility criteria | 6 | Specify study characteristics (e.g., PICOS, length of follow-up) and report characteristics (e.g., years considered, language, publication status) used as criteria for eligibility, giving rationale. | Methods (Systematic Review: Paragraphs 1 and 2) |
| Information sources | 7 | Describe all information sources (e.g., databases with dates of coverage, contact with study authors to identify additional studies) in the search and date last searched. | Methods (Systematic Review: Paragraph 1) |
| Search | 8 | Present full electronic search strategy for at least one database, including any limits used, such that it could be repeated. | Supplementary Appendix (Additional Details on the Methodology: Electronic Search Strategies) |
| Study selection | 9 | State the process for selecting studies (i.e., screening, eligibility, included in systematic review, and, if applicable, included in the meta-analysis). | Methods (Systematic Review: Paragraphs 2 and 3) |
| Data collection process | 10 | Describe method of data extraction from reports (e.g., piloted forms, independently, in duplicate) and any processes for obtaining and confirming data from investigators. | Methods (Systematic Review: Paragraph 3) |
| Data items | 11 | List and define all variables for which data were sought (e.g., PICOS, funding sources) and any assumptions and simplifications made. | Methods (Systematic Review: Paragraph 2) |
| Risk of bias in individual studies | 12 | Describe methods used for assessing risk of bias of individual studies (including specification of whether this was done at the study or outcome level), and how this information is to be used in any data synthesis. | Methods (Systematic Review: Paragraph 2) |
| Summary measures | 13 | State the principal summary measures (e.g., risk ratio, difference in means). | Methods (Systematic Review: Paragraph 4) |
| Synthesis of results | 14 | Describe the methods of handling data and combining results of studies, if done, including measures of consistency (e.g., I^2^) for each meta-analysis. | Methods (Systematic Review: Paragraph 4) |
| Risk of bias across studies | 15 | Specify any assessment of risk of bias that may affect the cumulative evidence (e.g., publication bias, selective reporting within studies). | Methods (Systematic Review: Paragraph 2) |
| Additional analyses | 16 | Describe methods of additional analyses (e.g., sensitivity or subgroup analyses, meta-regression), if done, indicating which were pre-specified. | Methods (Systematic Review: Paragraph 4) |
|  |  |  |  |
| **Results** |  |  |  |
| Study selection | 17 | Give numbers of studies screened, assessed for eligibility, and included in the review, with reasons for exclusions at each stage, ideally with a flow diagram. | Results (Characteristics of Included Studies: Paragraph 1); Figure 1 |
| Study characteristics | 18 | For each study, present characteristics for which data were extracted (e.g., study size, PICOS, follow-up period) and provide the citations. | Results (Characteristics of Included Studies: Paragraph 1); Table 1 |
| Risk of bias within studies | 19 | Present data on risk of bias of each study and, if available, any outcome level assessment (see item 12). | Not conducted (see Methods [Systematic Review: Paragraph 2]) |
| Results of individual studies | 20 | For all outcomes considered (benefits or harms), present, for each study: (a) simple summary data for each intervention group (b) effect estimates and confidence intervals, ideally with a forest plot. | Table 1 |
| Synthesis of results | 21 | Present results of each meta-analysis done, including confidence intervals and measures of consistency. | Results (Narrative Synthesis: Paragraphs 1 to 3) |
| Risk of bias across studies | 22 | Present results of any assessment of risk of bias across studies (see Item 15). | Not conducted (see Methods [Systematic Review: Paragraph 2]) |
| Additional analysis | 23 | Give results of additional analyses, if done (e.g., sensitivity or subgroup analyses, meta-regression [see Item 16]). | Results (Narrative Synthesis: Paragraphs 1 to 3) |
|  |  |  |  |
| **Discussion** |  |  |  |
| Summary of evidence | 24 | Summarize the main findings including the strength of evidence for each main outcome; consider their relevance to key groups (e.g., healthcare providers, users, and policy makers). | Discussion (Summary of Findings: Paragraph 1) |
| Limitations | 25 | Discuss limitations at study and outcome level (e.g., risk of bias), and at review-level (e.g., incomplete retrieval of identified research, reporting bias). | Discussion (Limitations: Paragraph 1) |
| Conclusions | 26 | Provide a general interpretation of the results in the context of other evidence, and implications for future research. | Discussion (Comparison with Previous Studies: Paragraphs 1; Interpretations and Implications: Paragraphs 1 to 2) |
|  |  |  |  |
| **Funding** |  |  |  |
| Funding | 27 | Describe sources of funding for the systematic review and other support (e.g., supply of data); role of funders for the systematic review. | Declarations (Competing Interests and Funding) |
